# F-18-Fluorodeoxyglucose Positron Emission Tomography/CT Effectively Identifying Source of Infection in a Patient With Multiple Dialysis Arteriovenous Fistula Access Points

**DOI:** 10.7759/cureus.8516

**Published:** 2020-06-08

**Authors:** James T Roberts, Javier Villanueva-Meyer, Samuel Bezold, Samuel O Krider, Quan D Nguyen

**Affiliations:** 1 Diagnostic Radiology, University of Texas Medical Branch, Galveston, USA; 2 Radiology, University of Texas Medical Branch, Galveston, USA; 3 Nuclear Medicine, University of Texas Medical Branch, Galveston, USA

**Keywords:** site of infection, av fistula, screening, diagnostic imaging, nuclear medicine, end stage renal disease, inflammation, fdg-pet/ct

## Abstract

Radiologic imaging techniques, such as F-18-fluorodeoxyglucose positron emission tomography/computerized tomography (FDG PET/CT), provide diagnostic value in a variety of diseases. In cases of suspected infection, FDG PET/CT can find areas of fluorodeoxyglucose metabolism, correlating with local acute inflammation. The following case involves a man with end-stage renal disease (ESRD), who presented with symptoms of infection and positive blood cultures with high suspicion of arteriovenous fistula as the source of infection. The patient also had two central lines that could be a site of infection. Concerns for patient’s persistent positive blood cultures necessitated FDG PET/CT to confirm site of infection. Confirming active infection and the source of infection guides therapeutic measures and eliminates concern for other disease etiologies common in patients with ESRD.

## Introduction

Inflammation is the process where the immune system responds to pathogens, toxic substances, damaged cells, or radiation. When the immune system responds to infection, the inflammatory response seeks to contain any damage [1]. Identifying inflammation radiologically can be done by finding areas of edema, contrast enhancement, organ damage as well as places with increased areas of white blood cells (WBCs) and glucose metabolism [2]. 

When there is uncertainty in identifying the sources of infection, imaging can be used to aid diagnosis. Both CT and MRI have shown reliable results for imaging changes in inflammation, but there are concerns regarding the reliability on finding early metabolic changes in pathology. Different nuclear medicine techniques, such as WBC-labeled imaging, gallium-67 (Ga-67) scanning, and three-phase technetium-99-m-methyl diphosphonate bone scanning, have all been routinely used with differing reliabilities [3]. Indium-111 labeled leukocytes were first used in the 1970s for a variety of infections, including fever of unknown origins (FUO), followed by imaging of soft tissues with Ga-67 citrate in the 1980s [4]. 

Patients with end-stage renal disease (ESRD) require vascular access for hemodialysis treatment. An arteriovenous (AV) fistula is the common and reliable method for hemodialysis access. Infections in AV fistulas can occur frequently from extended use of the access [5]. Second behind cardiovascular complications, infections in ESRD patients make up 15%-36% of all mortality and 20% of all admissions [6]. The native or prosthetic AV fistula is a site that needs proper evaluation in patients who present with signs and symptoms of infection or with an FUO. If there is no conclusive evidence from physical examination that the AV fistula is the source of infection, there should be explorations of other ways to search for diagnosis.

F-18-fluorodeoxyglucose (FDG) is a glucose analog that has increased uptake in monocytes and macrophages in areas of infection and inflammation. The nuclear imaging technique FDG positron emission tomography (FDG PET) can find areas of FDG accumulation in tissues that correlate with areas of inflammation [7]. A joint study from The Netherlands and Italy found that FDG PET/CT provides accurate diagnosis in cases of sarcoidosis and spondylodiscitis and could potentially aid in diagnosis in cases of inflammatory bowel diseases, rheumatoid arthritis, autoimmune pancreatitis, osteomyelitis, prosthetic joint infections, and fungal infections [8]. FDG PET/CT is able to find areas of high fluorodeoxyglucose metabolism, and thus there is overlap on the usability of the FDG PET/CT to specify inflammation versus acute infection.

## Case presentation

**Figure 1:**
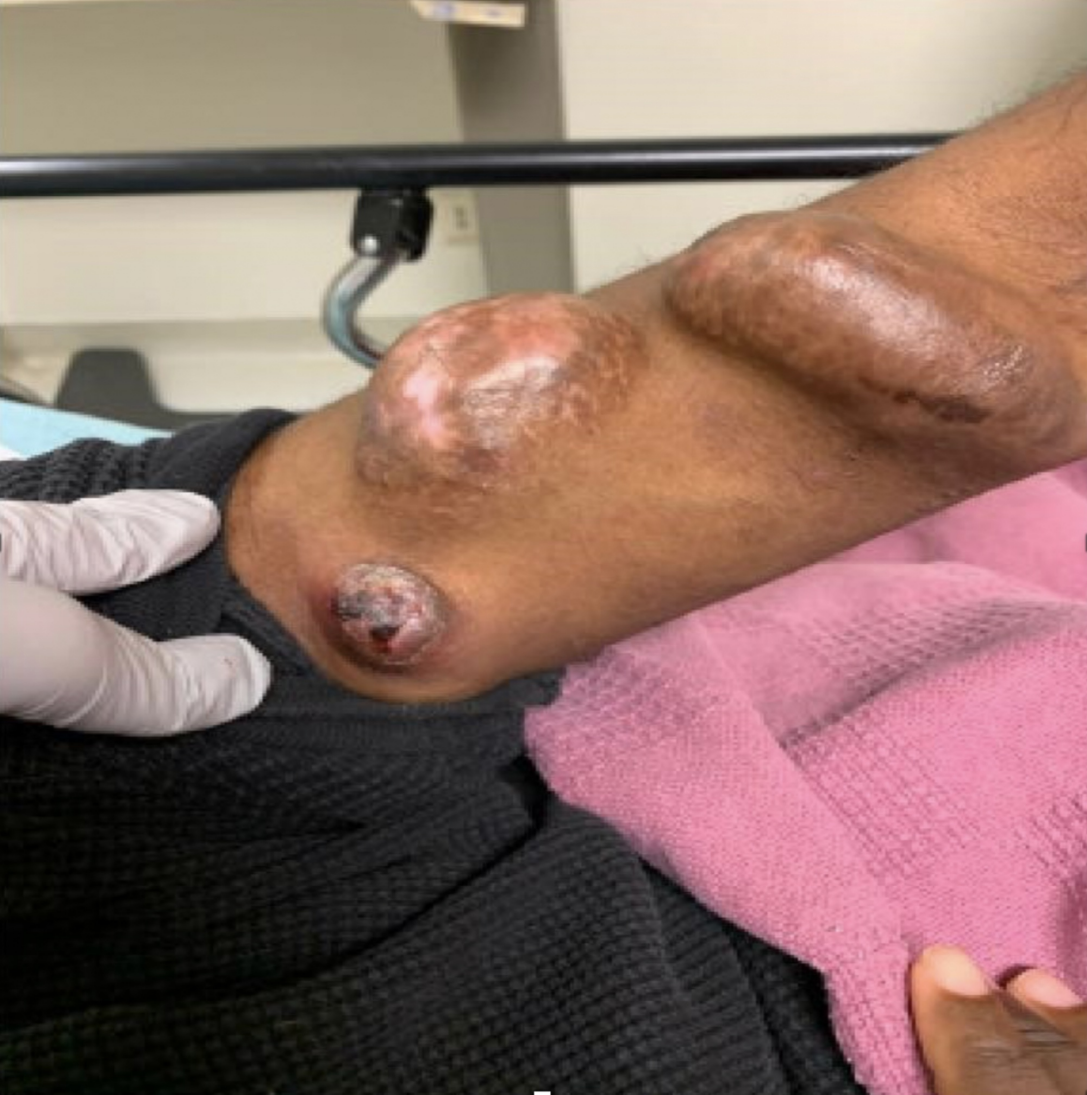
Initial Presentation of Left Arm Arteriovenous Fistula

A man with ESRD on hemodialysis presented to the emergency department (ED) with a history of fatigue, body aches, rigors, stomach pain, and chills. Two days prior to ED visit, he noticed “white pus” coming from his left arm fistula site as well as tenderness, scabbing, and crusting around the access site (Figure 1).

Routine physical exam revealed a left arm with tortuous AV fistula and scabbing and redness. The patient displayed other signs of infection including a 102.3°F fever and tachycardia. Ultrasound imaging of the left arm on admission revealed no evidence of abscess. The patient was admitted due to concerns of left fistula infection and started on antibiotics with follow-up cultures. 

The blood cultures were positive for methicillin-resistant Staphyloccous aureus (MRSA) bacteremia thought to be secondary to left arm AV fistula infection. The patient previously had a left internal jugular (IJ) fistula placed and a right arm AV fistula that were not currently used. While on vancomycin for infection five days post admission, the patient had persistent signs of bacteremia, including increased erythema and warmth around left arm AV fistula. Still highest on the differential diagnosis for source of infection was the AV fistula, and the patient was to continue antibiotic treatment for the bacteremia.

The attending team needed assurance that source of infection was from the left arm AV fistula and not the IJ or right arm fistula. Eleven days post admission, the patient had persistent bacteremia and given negative transthoracic and transesophageal echocardiogram; the infectious disease team recommended pursuing other diagnostic tests to confirm that the left arm fistula was the source of infection. 

**Figure 2:**
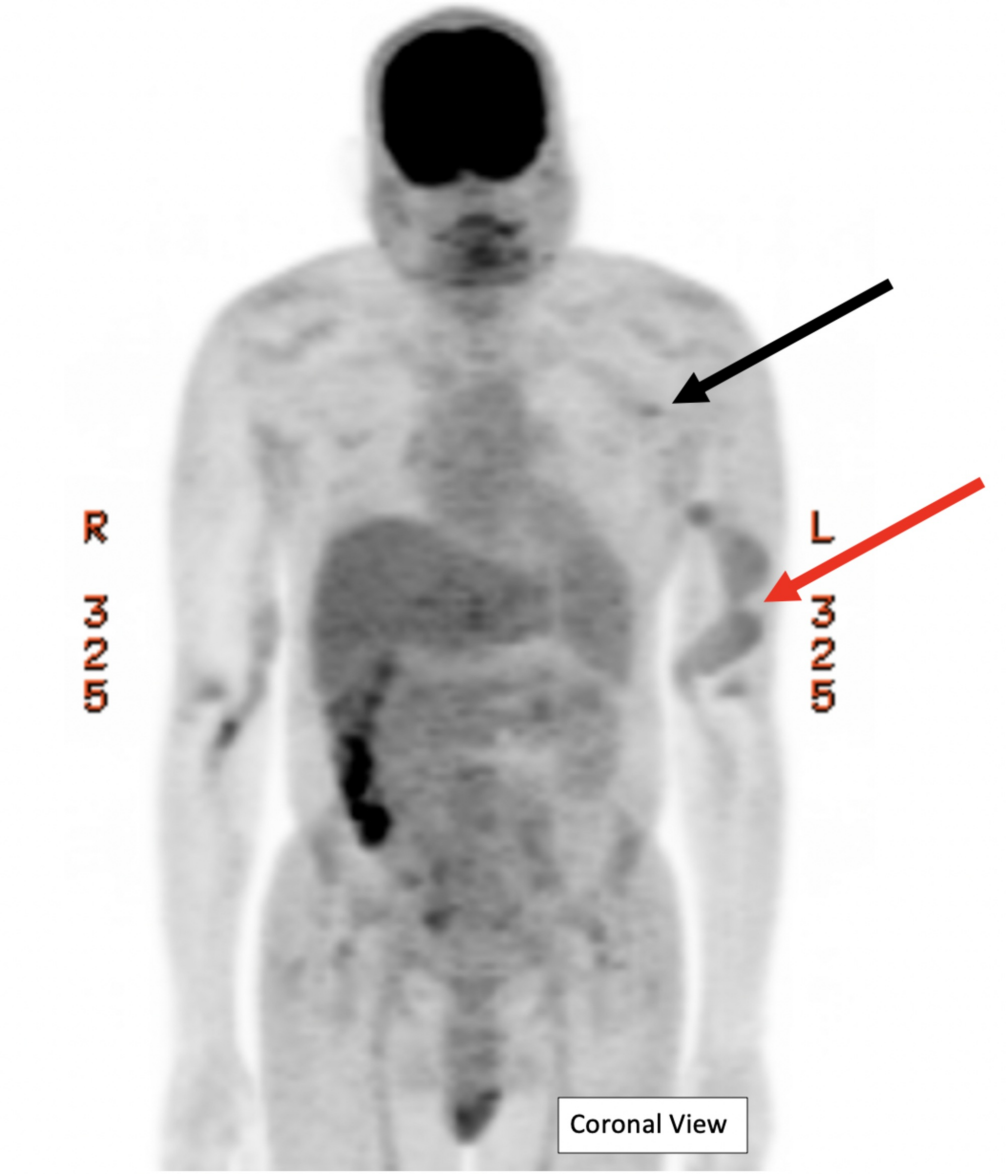
F-18-Fuorodeoxyglucose Positron Emission Tomography (FDG PET) Scan A diagnostic FDG PET scan found that the left upper extremity fistula had mild FDG uptake activity (red arrow) with standard uptake value (SUV) in the range of 1.7-2.1. The catheter extending to the left axilla (black arrow) shows moderate abnormal activity distal with SUV 2.1. There was no uptake at the left internal jugular fistula. Right arm fistula has mild activity. Physiologic activity noted in brain, salivary glands, blood pool, liver, and higher activity in colon, cecum, and testes.

**Figure 3:**
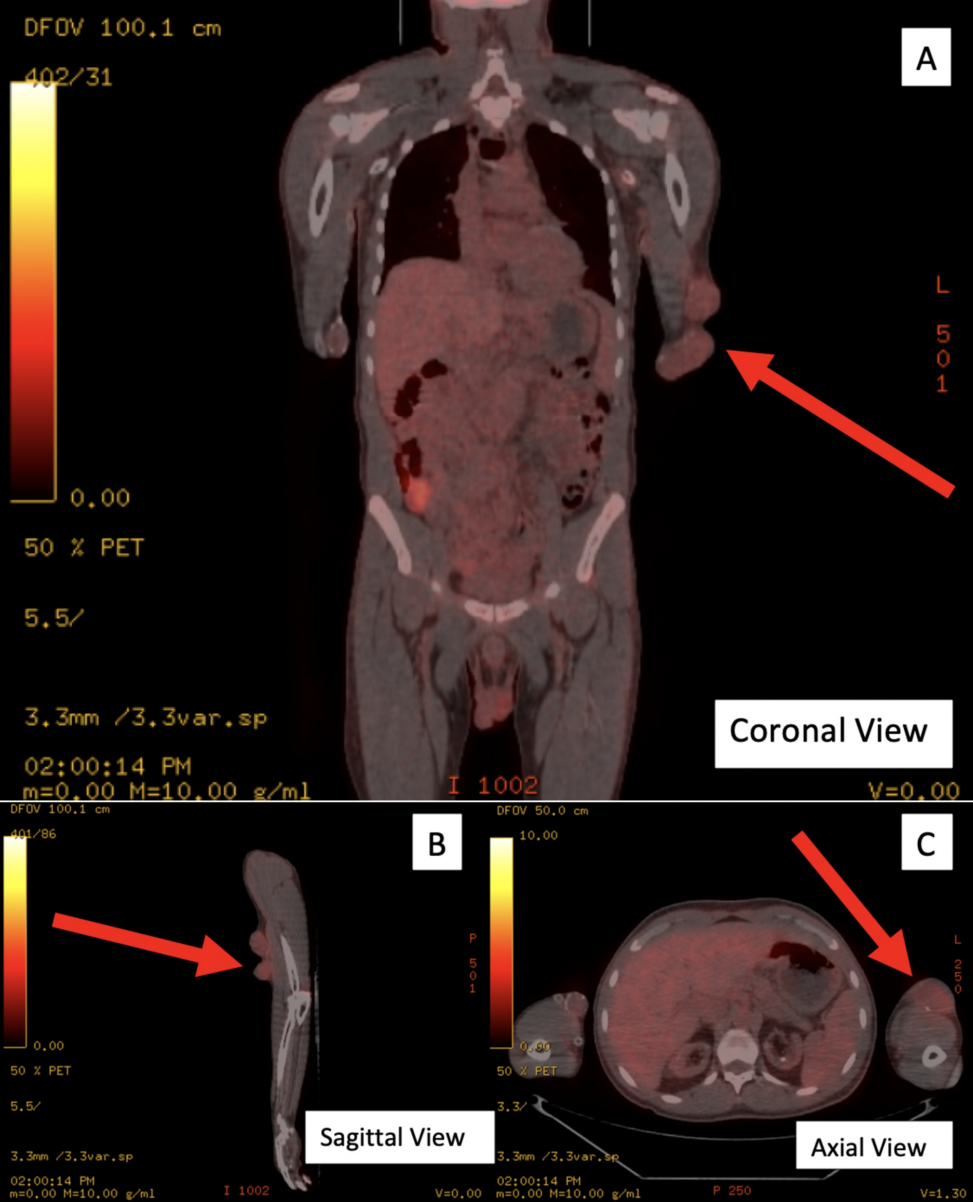
F-18-Fluorodeoxyglucose Positron Emission Tomography/Computerized Tomography (FDG PET/CT) Scan FDG PET/CT scan revealed abnormal FDG uptake suspicious for infection and inflammation at the left upper extremity fistula with highest suspicions at proximal subcutaneous. Scan includes coronal (A), sagittal (B), and axial (C) views. The red arrows denote site of increased FDG uptake.

To evaluate the source of infection, the patient received a FDG PET/CT scan 15 days after admission. The scan revealed inflammation in the left upper extremity fistula with highest suspicion at the proximal subcutaneous veins and at left axillary catheter (Figures 2, 3).

The vascular surgery team evaluated the arm after the FDG PET/CT scan and stated that there is an intraluminal clot that was causing a pressure build up. The patient underwent angioplasty followed by thrombectomy for resolution of the clot. The patient was determined to be stable for discharge with continued antibiotic therapy for six additional weeks with close monitoring by primary care physician, infectious disease, nephrology, vascular surgery, and endocrinology. 

## Discussion

Cases of persistent positive blood cultures require appropriate diagnostic techniques to find the source of the infection to guide treatment. Different ways of imaging that have been used include MRI and CT, which can detect structural changes including increased areas of edema and local tissue inconsistencies in contrast enhancements. Radiolabeled imaging techniques, including FDG PET scans, can detect infections more specific and often earlier than conventional MRI or CTs as molecular and cellular changes occur before edema and local organ damage can be visualized [9]. When inflammation is caused by an infection, it is paramount to have source of infection control to provide appropriate treatment. 

In a study of 33 patients who presented with suspicions of aortic prosthetic graft infections, PET scans showed a sensitivity of 91% compared to 64% in CT in detecting the presence of graft infection [10]. When focal uptake (versus diffuse) was set as the positive indicator, PET scan increased the specificity and positive predictive value to 95% [10]. Other studies have also reported that FDG PET with or without CT have had sensitivities more than 95% and specificities spanning from 75% to 95% in soft tissue infections [11]. 

In patients with FUO, FDG PET and other nuclear imaging techniques can be the differentiator in finding the source of infection. In one study on 20 patients with ESRD with FUO, FDG PET scans revealed areas of increased FDG uptake in 15 patients [12]. Of the 20 patients, 19 had a change in treatment to address the fever [12]. Gallium 67 citrate and labeled WBC scanning are often compared to FDG PET. FDG PET has been shown to diagnose a larger variety of illnesses outside of infection, including vasculitis, inflammatory bowel disease, sarcoidosis, and painless subacute thyroiditis [13]. FDG PET/CT has also shown to be more sensitive than Ga 67 citrate, and can be obtained in two hours versus two days with Ga 67 imaging [13,14]. FDG PET/CT is available at most hospitals for oncology imaging and the technique described for infection is identical. The cost of FDG PET/CT is over $100 compared to labeled WBC, which is over $1,000 [14]. 

Identifying the source of infection provides guidance to the appropriate treatment. Although there was high suspicion from initial presentation that left arm AV fistula was source of infections, cancer, rheumatic disease, vasculitis, sarcoidosis, and connective tissue diseases can all cause clinical signs similar to infection. In addition, this patient had two additional central lines that were potential sources of infections. FDG PET/CT provides high sensitivities to narrow differentials and provides insight to the presence and source of infection.

## Conclusions

FDG PET/CT correctly identified the site of infection in this patient with multiple dialysis AV access points. FDG PET/CT is readily available at most hospitals and is a faster exam compared to labeled WBC or Ga 67 citrate, while providing higher sensitivities than Ga 67 citrate. The cost of FDG PET is significantly lower than that of labeled WBC, and FDG PET/CT does not involve the handling of blood products. FDG PET/CT should be considered for cases of suspected infection where there is need for diagnosis or to ensure that therapeutic management is appropriate. FDG PET/CT imaging may play a role in emergent infection imaging in COVID-19 patients when multiorgan infection or inflammation is suspected.
